# Validating a molecular clock for nudibranchs—No fossils to the rescue

**DOI:** 10.1002/ece3.11014

**Published:** 2024-02-14

**Authors:** Kara K. S. Layton, Nerida G. Wilson

**Affiliations:** ^1^ School of Biological Sciences University of Aberdeen Aberdeen UK; ^2^ Department of Biology University of Toronto Mississauga Mississauga Ontario Canada; ^3^ Scripps Institution of Oceanography UC San Diego La Jolla California USA; ^4^ School of Biological Sciences University of Western Australia Crawley Western Australia Australia

**Keywords:** divergence rate, geminate pairs, molecular clock, Nudibranchia

## Abstract

Time calibrated phylogenies are typically reconstructed with fossil information but for soft‐bodied marine invertebrates that lack hard parts, a fossil record is lacking. In these cases, biogeographic calibrations or the rates of divergence for related taxa are often used. Although nudibranch phylogenies have advanced with the input of molecular data, no study has derived a divergence rate for this diverse group of invertebrates. Here, we use an updated closure date for the Isthmus of Panama (2.8 Ma) to derive the first divergence rates for chromodorid nudibranchs using multigene data from a geminate pair with broad phylogeographic sampling. Examining the species *Chromolaichma sedna* (Marcus & Marcus, 1967), we uncover deep divergences among eastern Pacific and western Atlantic clades and we erect a new species designation for the latter (*Chromolaichma hemera* sp. nov.). Next, we discover extensive phylogeographic structure within *C. hemera* sp. nov. *sensu lato*, thereby refuting the hypothesis of a recent introduction. Lastly, we derive divergence rates for mitochondrial and nuclear loci that exceed known rates for other gastropods and we highlight significant rate heterogeneity both among markers and taxa. Together, these findings improve understanding of nudibranch systematics and provide rates useful to apply to divergence scenarios in this diverse group.

## Graphical abstract

Here, for the first time, we derive divergence rates for nudibranchs using multigene data from a previously undetected geminate pair, with rates exceeding those reported for shelled gastropods. We also erect a new species designation for the Western Atlantic clade, *Chromolaichma hemera* sp. nov., and we uncover deep divergences within this clade.

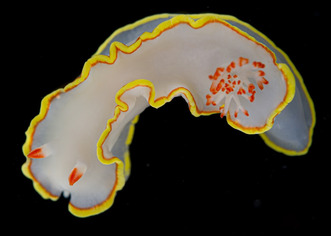

## 1 INTRODUCTION

There is no question that the concurrent estimation of divergence times with a phylogenetic tree improves inferences regarding evolutionary hypotheses (Hipsley & Muller, 2014; Ho, 2021; Landis, 2020; Marko, 2002). For many invertebrate groups, calibrating time‐trees with fossil information has been key to understanding rate variation across a range of lineages (e.g. Schultheiß et al., 2009; Wall‐Palmer et al., 2020). However, in many cases, fossil information is absent, scarce, or unreliable. This may be because the organisms live in habitats that rarely encounter fossilization or because they lack hard parts to fossilize, like many soft‐bodied marine organisms (Holland, 2016; Loeza‐Quintana et al., 2018). Alternative approaches to fossil calibrations include dating divergences using biogeographic calibrations, such as the Isthmus of Panama or the closure of the Bering Strait (e.g. Coppard et al., 2013; Hallas et al., 2016; Knowlton & Weigt, 1998; Loeza‐Quintana et al., 2018), but these approaches carry a number of assumptions. Most importantly, assigning dates to these events can be difficult to do with precision since dates often span a large period of time (Ho et al., 2015). However, recent work by O'Dea et al. (2016) demonstrated a gradual closure with the Isthmus of Panama forming 2.8 million years ago (Ma), a date that is most appropriate for shallow‐water organisms. Despite these advances, biogeographic calibrations can still be problematic when putative geminate species pairs are erroneously assigned and not recovered as sister‐species (Marko et al., 2015).

Soft‐bodied sea slugs such as nudibranchs, lack diagnostic hard parts large enough (and informative enough) to fossilize and this dearth of fossil data has hindered molecular clock calibration in the group. Although previous studies have calibrated nudibranch phylogenies with few geological data points (Hallas et al., 2016) or rates from other taxonomic groups (Lindsay et al., 2016), a lineage‐specific rate is lacking for nudibranchs. *Chromolaichma sedna*, a chromodorid nudibranch, is known on both sides of the Isthmus of Panama, but was first reported in the western Atlantic only in the 1980s (Bertsch, 1988). A subsequent surge in distribution records for this species in the western Atlantic in the 1990s led to support for a hypothesis of recent introduction in the Caribbean Sea (Goodheart et al., 2016). However, the remarkable conservation of external features in both eastern Pacific and western Atlantic populations (Bertsch, 1988), along with results from a broadly‐sampled molecular phylogeny of the Chromodorididae (Johnson & Gosliner, 2012), indicate that these allopatric populations are indeed conspecific and constitute a geminate pair. Investigation of the systematic relationship between these two geographic areas is critical to clarifying their taxonomy. Furthermore, the continued use of few diagnostic loci for nudibranch systematics and phylogenetics (e.g. Jung et al., 2020; Layton et al., 2018; Soong et al., 2020) justifies the need for deriving reliable evolutionary rates for these loci and taxa.

This study seeks to estimate rates of divergence for chromodorid slug species, based on a geminate pair with broad phylogeographic sampling, using the emergence of the Isthmus of Panama as a calibration point. This will provide the first estimate of divergence rates for fossil‐less, chromodorid nudibranchs. Next, we address the hypothesis that *C. sedna* is a recent introduction to the western Atlantic by investigating phylogeographic structure in the region. Finally, we revise the taxonomic status of the western Atlantic *Chromolaichma ‘sedna’*.

## 2 METHODS

### 2.1 Specimen collection and DNA sequencing

**FIGURE 1:**
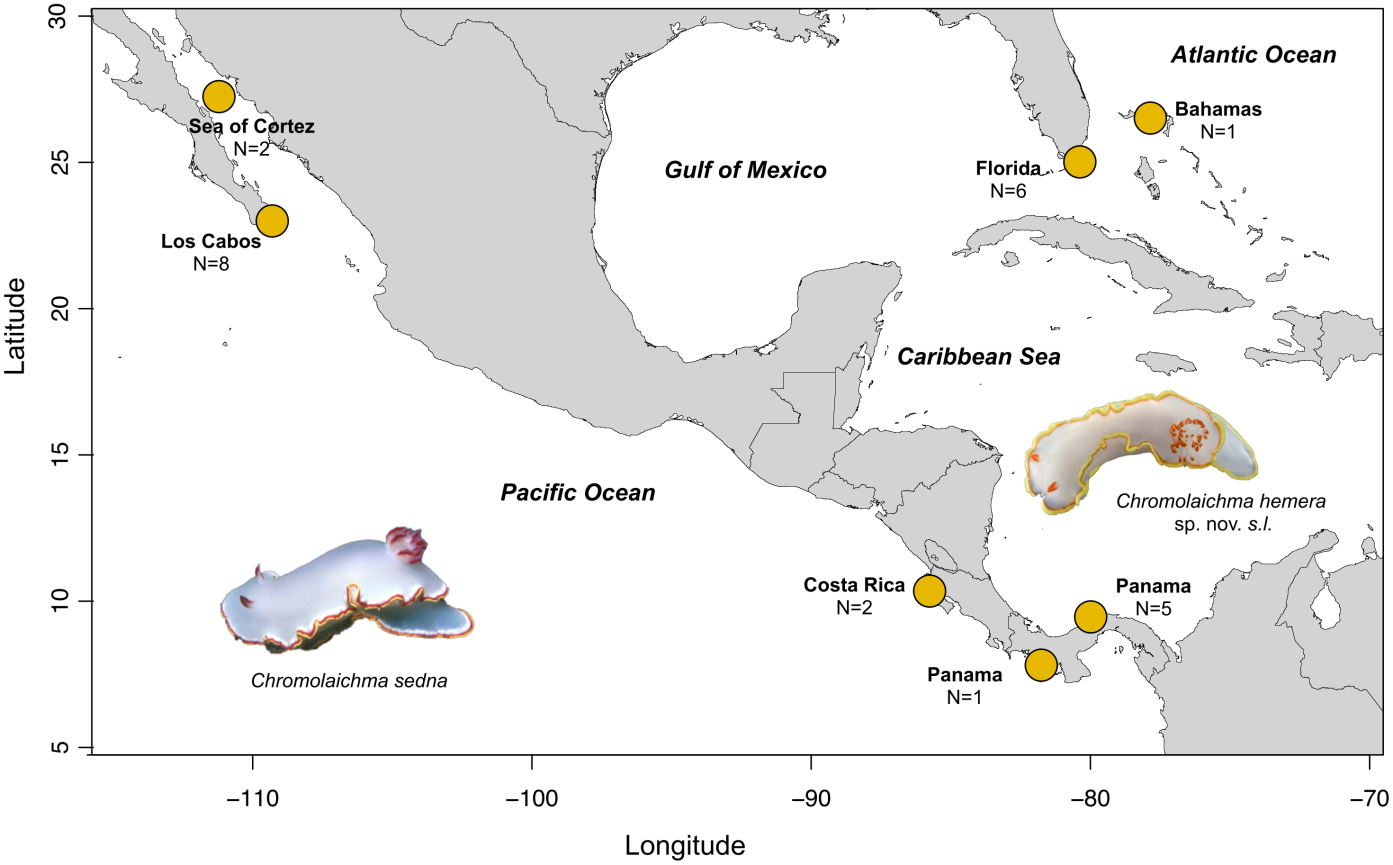
Sampling locations for 25 *Chromolaichma* specimens collected from either side of the Isthmus of Panama. Sample numbers appear next to locations. Images derived from iNaturalist (on left, photographer: S. & J. Johnson) and Museum of Comparative Zoology (on right, photographer: G. Giribet).

A total of 20 specimens of *C. ‘sedna’* were collected by hand on SCUBA at depths of 5–15 m at multiple sites in the Caribbean Sea and eastern Pacific Ocean, including near the type locality in the Sea of Cortez (Figure 1, Table 1). Two additional specimens were provided by Florida Natural History Museum and Museum of Comparative Zoology and sequence data for three individuals were sourced from Genbank (Table 1). All specimens were preserved in 96% ethanol in preparation for molecular analyses. Genomic DNA was extracted from a sample of the foot tissue using a DNeasy blood & tissue kit (Qiagen) following manufacturer's guidelines. PCR amplicons were purified using either Exo‐SapIT or gel purification. Two mitochondrial genes, cytochrome *c* oxidase subunit I (COI) and 16S ribosomal RNA (16S), and one nuclear gene, adenine nucleotide translocase (ANT), were targeted for sequencing. For PCR and sequencing, Folmer et al. (1994) primers were used for COI, Palumbi et al. (1991) primers were used for 16S, and Audzijonyte and Vrijenhoek (2010) primers were used for ANT. Amplicons were bidirectionally sequenced on an Applied Biosystems 3730 capillary sequencer. Forward and reverse sequences were edited and assembled in Sequencher (Gene Codes Corporation) and Geneious Prime 2021 (http://www.geneious.com/) and aligned with MAFFT (Katoh et al., 2002). A second alignment was trimmed to equal length in Geneious Prime 2021 for haplotype network construction.

**TABLE 1:** Specimen information for 25 *Chromolaichma ‘sedna’* samples collected in the Caribbean Sea and Pacific Ocean for this study, along with outgroup taxa.

Sample field ID	Registration number	Locality	Lat	Long	COI	16S	ANT
Western Atlantic							
pinnA1	SIO‐BIC M11636	Florida: Key Largo, Molasses Reef, The Pinnacles	25.0139° N	80.3708° W	EF535134	EF534062	PP133518
pinnA2	SIO‐BIC M18383	Florida: Key Largo, Molasses Reef, The Pinnacles	25.0104° N	80.3723° W	PP133106	PP135512	PP133514
wellB1	SIO‐BIC M18384	Florida: Key Largo, Molasses Reef, Wellwood Grounding	25.0105° N	80.3728° W	PP133107	PP135513	PP133515
wellB2	SIO‐BIC M18385	Florida: Key Largo, Molasses Reef, Wellwood Grounding	25.0105° N	80.3728° W	PP133108	PP135514	PP133516
pickering	SIO‐BIC M18386	Florida: Pickles Reef, Snapper ledge	24.9877° N	80.414° W	PP133109	PP135515	PP133511
NDR	FMNH‐UF‐411735	Florida: Keys, North Dry Rocks	25.0739° N	80.2817° W	PP133110	PP135511	PP133510
G354a	SIO‐BIC M18387	Panamá: Boca del Drago	9.4049° N	82.3251° W	PP133112	PP135517	PP133517
G354b	SIO‐BIC M18388	Panamá: Isla Bastimentos	9.4049° N	82.3251° W	PP133113	PP135520	PP133520
G357	SIO‐BIC M18389	Panamá: Isla Bastimentos	9.2853° N	82.1917° W	PP133114	PP135518	PP133512
G358	SIO‐BIC M18390	Panamá: Isla Bastimentos	9.2555° N	82.1252° W	PP133115	PP135519	PP133519
MCZ	MCZ381343	Panama: Isla Colón, STRI	9.3522° N	82.2569° W	PP133105	PP135500	‐
BAH	SIO‐BIC M13673	Bahamas: Sweetings Cay	26.6193° N	77.868° W	PP133111	PP135516	PP133513
Pacific							
SC02‐05	SIO‐BIC M18397	Sea of Cortez: Isla San Idelfonso	26.5747° N	111.4112° W	PP133116	PP135510	PP133528
CSLB	SIO‐BIC M18392	Mexico: Cabo San Lucas, Land's End	22.8782° N	109.8966° W	PP133117	PP135501	PP133525
SC09‐29	SIO‐BIC M18391	Sea of Cortez	‐	‐	PP133118	PP135502	‐
SMA	SIO‐BIC M18393	Mexico: San Jose del Cabo, Santa Maria	22.9154° N	109.8214° W	PP133122	PP135506	PP133522
SMB	SIO‐BIC M18394	Mexico: San Jose del Cabo, Santa Maria	22.9154° N	109.8214° W	PP133123	PP135505	PP133526
SMC	SIO‐BIC M18395	Mexico: San Jose del Cabo, Santa Maria	22.9154° N	109.8214° W	PP133124	PP135504	PP133524
SMD	SIO‐BIC M18396	Mexico: San Jose del Cabo, Santa Maria	22.9154° N	109.8214° W	PP133125	PP135503	‐
PelA	SIO‐BIC M13674	Mexico: Cabo San Lucas, Pelican Rock	22.8785° N	109.8988° W	PP133119	PP135509	PP133527
PelB	SIO‐BIC M13675	Mexico: Cabo San Lucas, Pelican Rock	22.8785° N	109.8988° W	PP133120	PP135508	PP133521
PelC	SIO‐BIC M13676	Mexico: Cabo San Lucas, Pelican Rock	22.8785° N	109.8988° W	PP133121	PP135507	PP133523
CASIZ175430*	CAS IZ175430	Costa Rica: Punta Carbon	10.3448° N	85.8639° W	JQ727877	JQ727758	‐
CASIZ175435*	CAS IZ175435	Costa Rica: Playa Real	10.3877° N	85.8388° W	JQ727879	JQ727759	‐
CASIZ175446*	CAS IZ175446	Panama: Golfo de Chiriquí, Isla Ulva	7.3091° N	81.7882° W	JQ727878	JQ727760	‐
Outgroups							
*Doriprismatica atromarginata*	CAS IZ177237	Philippines: Caban Island	13.6911° S	120.8414° E	JQ727864	JQ727746	‐
*Tyrinna evelinae*	SIO‐BIC M18401	Mexico: Isla San Esteban	28.7202° N	112.6119° W	‐	‐	PP236067

*Note*: GenBank accession numbers are provided for COI, 16S and ANT. An asterisk marks samples that derive from GenBank and a hyphen denotes missing information.

### 2.2 Phylogenetic analyses and species delimitation

Both individual gene and concatenated gene datasets were used for constructing maximum likelihood (ML) trees in RAxML (Stamatakis, 2006) implemented in the raxmlGUI v2.0 (Edler et al., 2020). Trees were built with a GTR + G model and 1000 bootstrap replicates with *Doriprismatica atromarginata* as an outgroup for the concatenated dataset, COI and 16S, and with *Tyrinna evelinae* as an outgroup for ANT. Total branch length (TBL) of each gene tree was summed and used to derive a mutation rate ratio for mitochondrial (μm) and nuclear loci (μn) following Duda (2021). For μm, an average TBL of both genes was used. Both the Assemble Species by Automatic Partition (ASAP) algorithm (Puillandre et al., 2021) and the Bayesian bPTPmodel (Zhang et al., 2013) were used to partition the COI dataset into unique genetic clusters using default parameters. A minimum spanning haplotype network was generated with trimmed COI data using the haploNet function in the *pegas* package in R (Paradis, 2010).

### 2.3 Estimating divergence rates

Pairwise distances were calculated for COI, 16S, and ANT in MEGAX (Kumar et al., 2018) using a maximum composite likelihood model and pairwise deletion. To calculate a rate of divergence for this species, we used the minimum sequence divergence between geminate pairs and a closing date of the Isthmus of Panama of 2.8 mya (O'Dea et al., 2016). We chose to use the more conservative minimum sequence divergence rather than mean divergence to prevent the inclusion of accumulated intraspecific changes. Rates of divergence were calculated for all genes. The substitution rate is presented as half of the divergence rate. To determine whether the data were evolving in a clock‐like fashion, a maximum likelihood clock test was conducted in MEGAX (Kumar et al., 2018) using a Tamura‐Nei model and rate heterogeneity for complete COI, 16S and ANT sequences. Divergence rates from other geminate pairs of marine invertebrates were retrieved from the literature for comparative purposes, along with relevant life history information. Life history information, including lifespan, reproductive mode and larval dispersal, were obtained for the genus where possible but may represent broader taxonomic patterns in some instances.

## 3 RESULTS

### 3.1 Deep divergence within and among ocean basins

A total 22 COI, 22 16S and 19 ANT sequences were generated for *C. ‘sedna’* in this study, with 25 total sequences of COI and 16S including those sourced from GenBank. Data for these specimens and GenBank accession numbers are provided in Table 1. The final COI alignment was 658 bp in length, the 16S alignment was 459 bp and the ANT alignment was 553 bp. A trimmed COI alignment of 590 bp was used for the haplotype network. Mean intraspecific divergence of *C. ‘sedna’* was 8.7% (range 0.0%–15.7%) at COI, 3.9% (range 0.0%–8.1%) at 16S and 0.3% (range 0.0%–0.7%) at ANT (Tables S1–S3).

**FIGURE 2:**
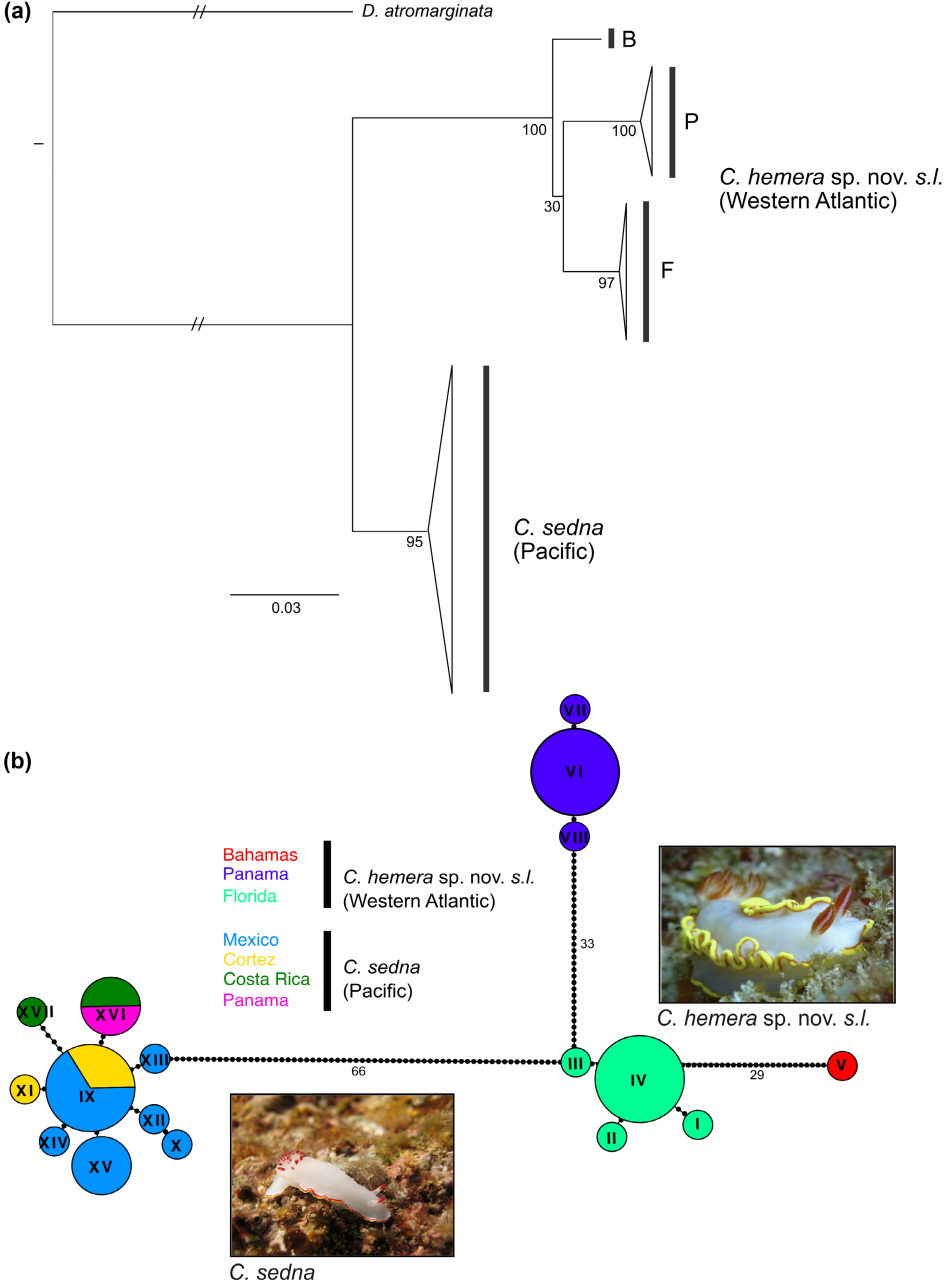
(a) Maximum‐likelihood phylogeny (COI + 16S + ANT) for *Chromolaichma ‘sedna’* where triangles represent collapsed clades, hash marks indicate the branch was shortened by 50%, and bootstrap support is presented at nodes. Individual bars represent congruent clades from the ABGD and bPTP delimitation analyses. (b) COI haplotype network where color corresponds to geographic location and the size of circles corresponds to sample size. Roman numerals are assigned to unique haplotypes and black circles denote mutational steps. Images derive from iNaturalist and represent *C. sedna* and *Chromolaichma hemera* sp. nov. lineages from Baja California and Chetumal, Mexico (photographers: on left, A. Velasco and on right, María Ecléctica Photography).

The COI + 16S + ANT ML phylogeny showed that western Atlantic and eastern Pacific *C. ‘sedna’* formed separate clades, with more structure in the western Atlantic than in the eastern Pacific (Figure 2a). These same patterns were recovered in individual gene trees (Figure S1). Three western Atlantic clades were strongly supported in the combined phylogeny (BP > 90), although support at interior nodes was weak (Figure 2a). These results were reinforced by the species delimitation analyses that partitioned western Atlantic individuals into three clusters, corresponding to geographic region (Bahamas, Panama, and Florida) (Figure 2a). The minimum divergence between individuals in the western Atlantic and eastern Pacific was 13.6% at COI, 5.7% at 16S and 0.5% at ANT. Within the western Atlantic clade mean intraspecific divergence at COI was 4.0%, with a mean divergence of 5.4% between Florida and Bahamas, 6.5% between Bahamas and Panama, and 6.5% between Florida and Panama. The minimum sequence divergence at COI among these clades were 5.4%, 6.3%, and 6.2%, respectively. Within the eastern Pacific clade mean intraspecific divergence at COI was 0.45%.

The same patterns were also recovered in the haplotype network analysis, with different ocean basins showing significant differentiation, as well as regions within the western Atlantic showing deep haplotype divergence and no shared haplotypes (Figure 2b). In total, 8 and 9 unique haplotypes were identified from 12 and 13 western Atlantic and eastern Pacific individuals, respectively (Figure 2b). Within the Pacific, all haplotypes were closely related to the central haplotype in Mexico and the Sea of Cortez.

### 3.2 Rate variation

Given a minimum sequence divergence of 13.6% among *C. ‘sedna’* individuals on either side of the Isthmus of Panama, and an estimated age of separation of 2.8 million years, COI is expected to diverge at a rate of 4.9% per million years. For 16S, with a minimum sequence divergence of 5.7%, this rate was 2.0% per million years. For ANT, with a minimum sequence divergence of 0.5%, this rate was 0.2% per million years. Divergence rates of 4.9%, 2.0%, and 0.2% correspond to substitution rates of 2.5% for COI, 1.0% for 16S, and 0.1% for ANT. COI and 16S rates are 25 and 10 times higher than the ANT rates, respectively. This variation in divergence rate also underpins differences in pairwise distance among loci (Figure S2). Total branch length of the COI, 16S, and ANT trees was 0.299, 0.136, and 0.006, respectively, with a μm/μn value of 39.5. The clock test was insignificant for COI, 16S, and ANT (*p* = .9, *p* = .7, *p* = .06), indicating that the null hypothesis of equal evolutionary rates throughout the tree could not be rejected (Table 2).

**TABLE 2:** COI divergence rates (% per myr) across geminate pairs of marine invertebrates separated by the Isthmus of Panama.

Taxonomy (Class: Order)	COI rate (%/myr)	Number of species	Lifespan	Reproductive mode	Larval dispersal	References
Mollusca						
Bivalvia						
Arcidae	1.2%	301	Up to 25 y	Gonochoric & sequential hermaphrodism	Planktotrophic (↑)	Breton et al. (2017), Marko (2002), Moran (2004), Puljas et al. (2015), Zeinalipour et al. (2014)
Gastropoda						
Hydrobiidae: *Peringia*	2.6%	1 (1897)	1–2 y	Gonochoric	Planktotrophic (↑)	Barnes (1990), Brownlow et al. (2008), Hershler and Davis (1980), Wilke (2003)
Tegulidae: *Tegula*	2.4%	48 (66)	Up to 25 y	Gonochoric	Lecithotrophic (↓)	Gallivan and Danforth (1999), Hellberg and Vacquier (1999), Kulikova and Omel'yanenko (2000), Moran (1997)
Chromodoridiidae: *Chromolaichma*	4.9%	3 (392)	1 y	Simultaneous hermaphrodism	Planktotrophic (↑)	Aerts (1994), Rudman (2008), This study
Plakobranchidae: *Elysia*	4.0%	102 (136)	Up to 1 y	Simultaneous hermaphrodism	Poeciligonous (↑ ↓) (planktotropic & lecithotrophic)	Clark (1975), Ellingson and Krug (2016)
Annelida						
Polychaeta						
Alvinellidae: *Alvinella*	2.4%	2 (11)	2.7 y	Gonochoric	Lecithotrophic (↓)	Chevaldonné et al. (2002), Desbruyères et al. (1998), Hilário et al. (2015), McHugh and Fong (2002), Wilke et al. (2009)
Arthropoda						
Malacostraca						
Alpheidae: *Alpheus*	1.4%	321 (1119)	1.2 y	Gonochoric	Lecithotrophic (↓)	Knowlton and Keller (1986), Knowlton and Weigt (1998), Mossolin et al. (2006), Pavanelli et al. (2008)
Penaeidae: *Penaeus*	4.3%	33 (242)	1–2 y	Gonochoric	Lecithotrophic (↓)	Baldwin et al. (1998), Champion (1987), Voltolina and Angulo (2007)
Echinodermata						
Echinoidea						
Cidaridae: *Eucidaris*	2.8–3.7%	12 (387)	4–5 y	Gonochoric	Planktotrophic (↑)	Lessios et al. (1999), McPherson (1965)
Asteroidea						
Oreasteridae: *Oreaster*	5.0%	2 (63)	Up to 7 y	Gonochoric	Planktotrophic (↑)	Hart et al. (1997)

*Note*: The number of species per taxonomic group was sourced from WoRMS, based on accepted records only, and are presented for both the genus and family (in brackets) where applicable. Information about th lifespan, reproductive mode, and larval dispersal were obtained for the genus where possible but may represent broader taxonomic patterns in some instances. Arrows indicate high or low larval dispersal potential and “y” represents the year.

### 3.3 Systematics

Chromodorididae Bergh, 1891

*Chromolaichma* Bertsch, 1977

Type *Casella sedna* Ev. Marcus & Er. Marcus 1967

**FIGURE 3:**
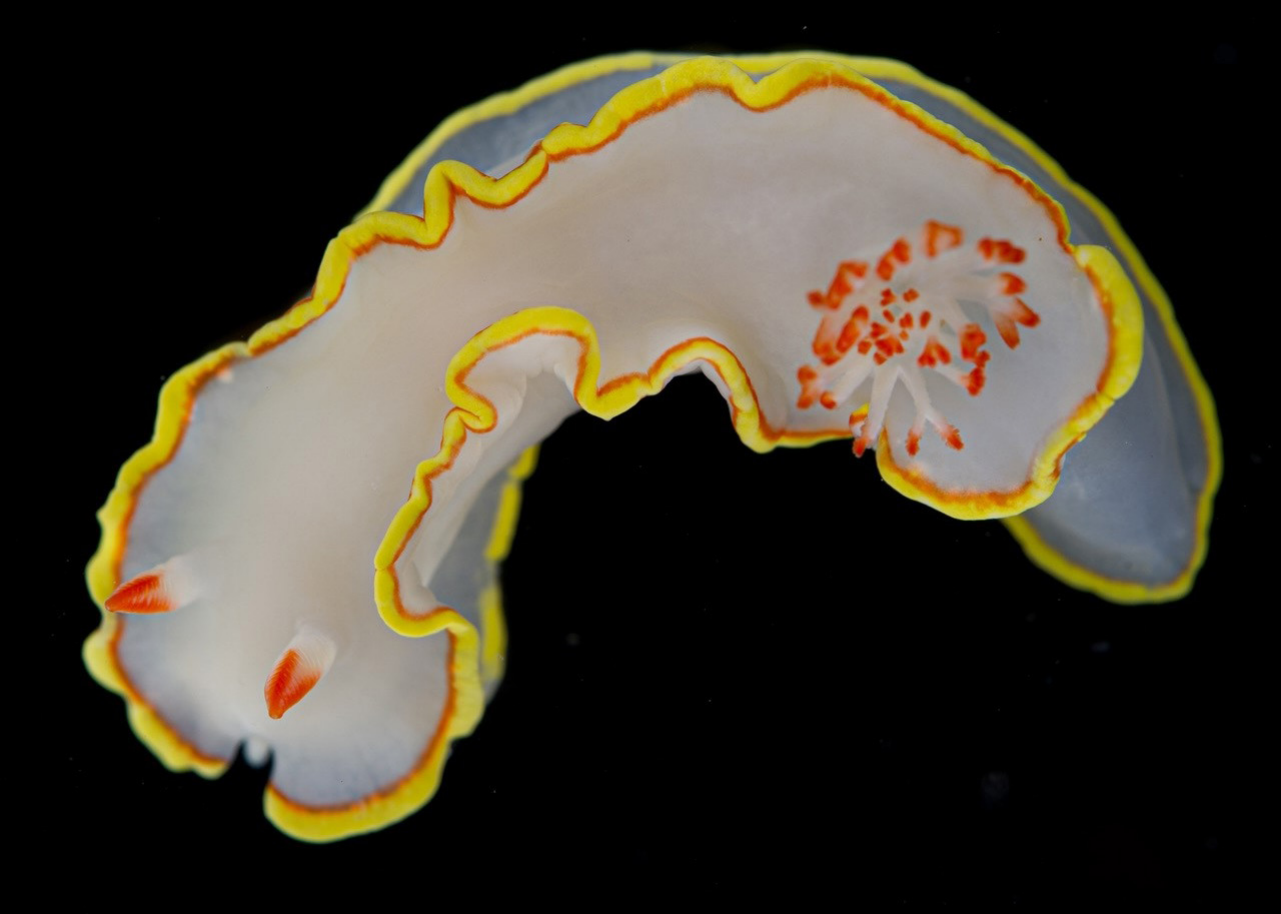
*Chromolaichma hemera* sp. nov. holotype collected from Smithsonian Tropical Research Station (STRI) (MCZ:M:381343) (photographer: G. Giribet).

*Chromolaichma hemera* sp. nov. (Figures 1, 2, 3).

*Zoobank registration*. LSID: 42E0650E‐C2F3‐4A2E‐A993‐2CED24D17F82

*Chromolaichma sedna*—Matsuda & Gosliner 2018 (in part).

*Dorisprismatica sedna*—Johnson & Gosliner 2012 (in part), Goodheart et al. 2016.

*Glossodoris sedna*—Bertsch 1988, Lyons 1989, Humann & Deloach 2002, Debelius & Kuiter 2007 (in part), Behrens & Hermosillo 2005 (in part), Valdés et al. 2006.

#### 3.3.1 Holotype

MCZ:M:381343, Smithsonian Tropical Research Station (STRI), Isla Colón, Archipiélago de Bocas del Toro, Panamá, 9.35215000°, −82.256990°, Caribbean Sea. Collected: 2014‐03‐19/2014‐03‐23. Coll: G. Giribet [https://mczbase.mcz.harvard.edu/guid/MCZ:Mala:381343].

#### 3.3.2 Paratype

SIO‐BIC M18386, Snapper Ledge 1, Pickles Reef, Key Largo, Florida, USA, 24.9877° N, 80.414° W, Western Atlantic. Collected: 2005‐10‐05, 5 m. Coll: N. Wilson.

#### 3.3.3 Etymology

Named after the Greek goddess of the day, Hemera.

#### 3.3.4 Distribution

Gulf of Mexico (Arrecife Alacranes), North Atlantic (Florida, The Bahamas) and the Caribbean Sea (Mexico, Belize, Honduras, Costa Rica, Panama, Turks and Caicos, Dominican Republic, Saint‐Barthélemy, Caribbean Netherlands). Not reported on the continental South American coastline.

#### 3.3.5 Description

Body soft, elongate and translucent white, with some specimens (including the holotype) with some orange–red dusting on the dorsum and sides of foot. Mantle edge usually crenulated. Tail extends beyond mantle. Mantle with yellow marginal band, and thinner red submarginal band. Rarely, an opaque band sits inside the red submarginal band. Similar bands of color appear on the foot margin, although the red submarginal band can be very thin or absent. Rhinophore stalks, translucent white. Lower part of the rhinophore clubs are translucent white, with anterior top third lamellae red. For most specimens, posteriorly, the stem that the rhinophore lamellae are attached to is red, and anteriorly, white. Lamellae evenly red around the upper part of rhinophores. Some specimens show lateral rhinophoral lamellae as white. Simple pinnate gills form a double spiral, and wriggle rhythmically. Large specimens may be bipinnate at tips. Distal tips of outer rachis and lamellae are red.

Radular described fully in Bertsch (1988). In short, the radular formulae for two specimens from Tavenier Key were 133 × 54.1.54 and 128 × 49.1.49 (LACM 83–147 and CASIZ 064510, respectively). The central rachidian tooth is a small, reduced, triangular tooth. The innermost tooth in each row had 2–4 denticles on the inner side and 3–5 outer side denticles. The rest of the teeth in each half row have 4–8 denticles on the outer side, but these became smooth for the outermost 10–20 teeth. The jaw rodlets were mostly bifid.

The reproductive system of these two specimens were examined by Bertsch (1988), who stated that the morphology of the penis, vas deferens, and the arrangement and relative size of the vagina, insemination duct, bursa copulatrix and the receptaculum seminis were all identical to the holotype of *C. sedna* (Marcus & Marcus, 1967).

#### 3.3.6 Remarks

*Chromolaichma* was only recently resurrected by Matsuda and Gosliner (2018) to encompass three species (*C. dalli*, *C. edmundsi*, *C. sedna*). In previous family‐level revisions, Rudman (1984) considered *Chromolaichma* as a synonym of *Glossodoris*, while Johnson and Gosliner (2012) placed it in ‘*Doriprismatica*’, later referred to as *Doriprismatica*.

**FIGURE 4:**
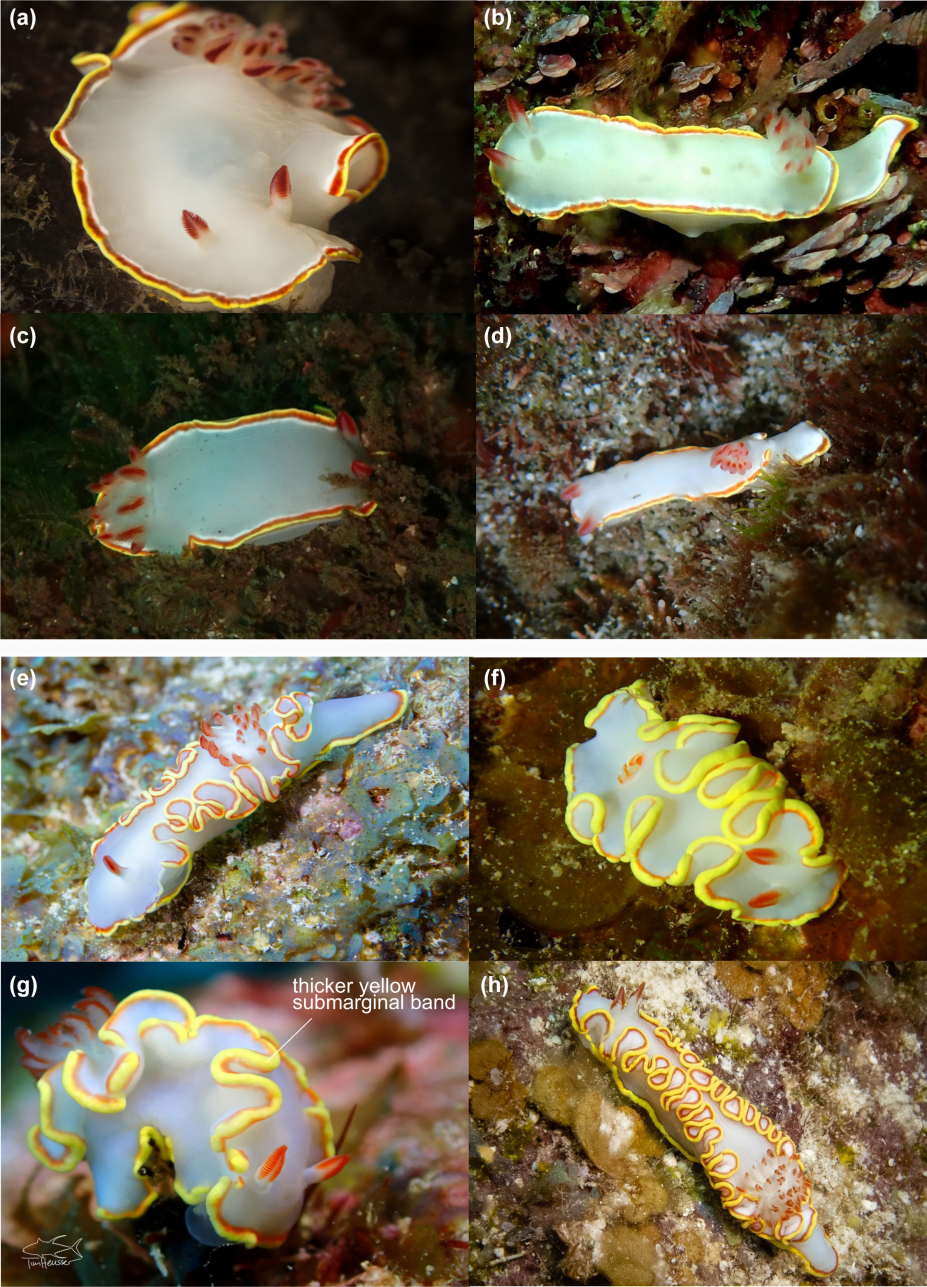
Images from iNaturalist representing *Chromolaichma sedna* (a–d) and *Chromolaichma hemera* sp. nov. (e–h) illustrating key differences in the color and crenulation of the mantle edge. *C. sedna* from (a, b) Mexico (photographers: V. Mas and M. Krampf), (c) El Salvador (photographer: A. Trejo), and (d) Costa Rica (photographer: K. Soto Venegas). *C. hemera* sp. nov. from (e) Florida (photographer: A. Shure), (f) Bahamas (photographer: M. Bokach), and (g, h) Belize (photographers: T. Heusse and A. McKinlay).

Morphological conservation between *C. hemera* sp. nov. and *C. sedna* was noted by Bertsch (1988) who described them as nearly identical, noting slight differences in background color (not as white as *C. sedna*) and with possibly more crenulations in the mantle (Figure 4). *Chromolaichma hemera* sp. nov. also exhibits a wider yellow submarginal band than *C. sedna* (Figure 4). Bertsch noted that the reproductive system was identical to that illustrated in the original description of *C. sedna* (Marcus & Marcus, 1967) and that the radular morphology matched the holotype, and fell within the range for specimens described from the Gulf of California (Bertsch, 1978). Overall, he did not consider these slight morphological differences to warrant a new species, but the addition of molecular data that demonstrates deep genetic divergences does support the establishment of a new species for the western Atlantic and Caribbean specimens. Molecular data also demonstrates deep divergences among western Atlantic and Caribbean clades, which may represent additional cryptic species. A lack of geographically intermediate samples and additional morphological data prevents their description here, so for now, we refer to all lineages in the western Atlantic and Caribbean clades as *C. hemera* sp. nov. sensu lato. Upon interrogation of publicly available images, there are notable differences in rhinophore colouration between individuals in the lesser Antilles and the rest of the Caribbean, but additional work is needed to verify this preliminary finding. Finally, the name *C. sedna* is now restricted to specimens in the Gulf of California and the eastern Pacific.

## 4 DISCUSSION

### 4.1 A new species in the western Atlantic and deep divergences within populations

This study uncovered strong geographic structuring of *C. ‘sedna’*, with reciprocal clades in the western Atlantic and eastern Pacific, providing support for a previously unrecognized geminate species pair. The minimum sequence divergence among individuals on either side of the Isthmus (13.6% at COI) is much greater than intraspecific divergences reported for other nudibranchs (0%–6%) (e.g. Fritts‐Penniman et al., 2020; Korshunova et al., 2018; Layton et al., 2018), and molluscs more generally (0%–5%) (e.g. Layton et al., 2014), and it aligns with divergences in other geminate species (e.g. 6.3%–26.5% in arcid bivalves, Marko & Moran, 2009; 8.9% in decapods, Pileggi et al., 2014). The monophyly of western Atlantic and eastern Pacific clades was strongly supported in the phylogenetic analyses and diagnostic morphological features set them apart. For instance, there are notable differences in the color of the marginal and submarginal bands and there are more crenulations in the mantle of the western Atlantic clade. Together, this evidence supports their status as separate species and a new name is established here for the western Atlantic clade (*C. hemera* sp. nov.). The discovery of a new species of nudibranch adds to a recent body of work employing molecular data to propel species discovery in this diverse group of invertebrates (e.g. Do et al., 2020; Epstein et al., 2019; Matsuda & Gosliner, 2018), but the nudibranch fauna of the western Atlantic has been less well studied than the Indo‐Pacific.

Our study also uncovered significant population structure within western Atlantic *C. hemera* sp. nov., as evidenced by deep haplotypic divergence among populations, negating the hypothesis that this species was recently introduced to the area through ship transport (e.g. Goodheart et al., 2016; Humann & Deloach, 2002). Bertsch (1988) mistakenly reported the location for a specimen shared with him by Lyons when the first tropical western Atlantic specimens were reported (see Lyons, 1989 for correction). Thus, Bertsch incorrectly reported that all known specimens were from a restricted locality, initiating the idea that they may represent an introduction, although not explicitly stating that. The earliest known specimens in the western Atlantic were identified from the Bahamas in 1978 (Lyons, 1989), only 11 years after the description of the eastern Pacific species *C. sedna*. The deep molecular divergences among tropical western Atlantic populations do not support a recent introduction scenario and instead may represent a cryptic species complex (*C. hemera* sp. nov. *sensu lato*). Conversely, we uncovered a lack of structure within the eastern Pacific clade. Although surprising, this has also been shown in *Tripneustes* sea urchins (Lessios et al., 2003) and the pronghorn spiny lobster (*Panulirus penicillatus*) (Chow et al., 2011) and it may be due to differences in oceanographic currents, habitat complexity and glacial sea level changes in the region.

There are several possible explanations for the deep divergence among western Atlantic clades. First, these deep divergences might reflect missing taxa given the high extinction rate in the region following the closure of the Isthmus (Lessios, 2008; Marko et al., 2015), or they might reflect rapid diversification following the split (Thacker, 2017). The discovery of a distinct genetic clade at each region in the western Atlantic (e.g. Panama, Bahamas and Florida) is congruent with patterns in other marine invertebrates in the area. For instance, DeBiasse et al. (2016) uncovered deep divergences among populations of the brooding sponge, *Callyspongia vaginalis*, with isolation among Central American and Caribbean clades and a further phylogeographic break between populations in Florida and the Bahamas. Alternatively, deep sequence divergences may be the result of accelerated rates of evolution. This is a particularly plausible scenario for nudibranchs given that the COI divergence rate derived here (4.9% per myr) is significantly higher than rates reported in other (shelled) gastropods, although very similar to rates in other shell‐less gastropods (e.g. *Elysia*, Ellingson & Krug, 2016).

### 4.2 Rate heterogeneity among geminate lineages

Rate heterogeneity has been invoked to explain variation in sequence divergence observed across marine geminate pairs (Marko, 2002), but this is the first study to summarize these rates. Here, we found significant rate heterogeneity among marine invertebrate genera that share a similar lifespan, reproductive mode and dispersal potential, suggesting that life history traits do not drive differences in mitochondrial rates. These findings align with recent work by Saclier et al. (2018), who found that while nuclear substitution rates were linked to generation time in isopods, mitochondrial substitution rates were independent of life history traits. Some of the seminal work linking mutation rates to life history traits was focused on only a few taxa and loci (e.g. Müller & Albach, 2010), thereby limiting our understanding of rate variation across the genomes of most species. In fact, we uncovered significant differences in nuclear and mitochondrial substitution and mutation rates, as evidenced by an elevated μm/μn ratio, similar to findings in other gastropods (Duda, 2021). Given the evidence for rate variation among both taxa and loci, future work will look to reassess substitution rates using transcriptome data from recent phylogenomic studies (Layton et al., 2020; Moles & Giribet, 2021). However, because COI, 16S, and ANT remain some of the most commonly used genes in species‐level nudibranch investigations, the lineage‐specific rates presented here will help improve understanding of divergence scenarios in this diverse group.

## AUTHOR CONTRIBUTIONS

**Kara K. S. Layton:** Conceptualization (supporting); formal analysis (lead); investigation (equal); validation (equal); visualization (equal); writing – original draft (equal); writing – review and editing (equal). **Nerida G. Wilson:** Conceptualization (lead); formal analysis (supporting); funding acquisition (lead); investigation (equal); resources (lead); validation (equal); visualization (equal); writing – original draft (equal); writing – review and editing (equal).

## CONFLICT OF INTEREST STATEMENT

The authors declare that there are no conflicts of interest.

## Supporting information


Appendix S1

## Data Availability

All sequence data are available on GenBank (accession numbers are provided in Table 1) and gene alignments can be retrieved from https://doi.org/10.5061/dryad.ns1rn8q08. Tissue samples and DNA extractions are vouchered at Scripps Institution of Oceanography, Florida Museum of Natural History and the Museum of Comparative Zoology.
