# Supplementary material for: Validating a molecular clock for nudibranchs—No fossils to the rescue
Source: Ecol Evol. 2024 Feb 14;14(2):e11014. doi: 10.1002/ece3.11014 (PMC10867498; doi:10.1002/ece3.11014)

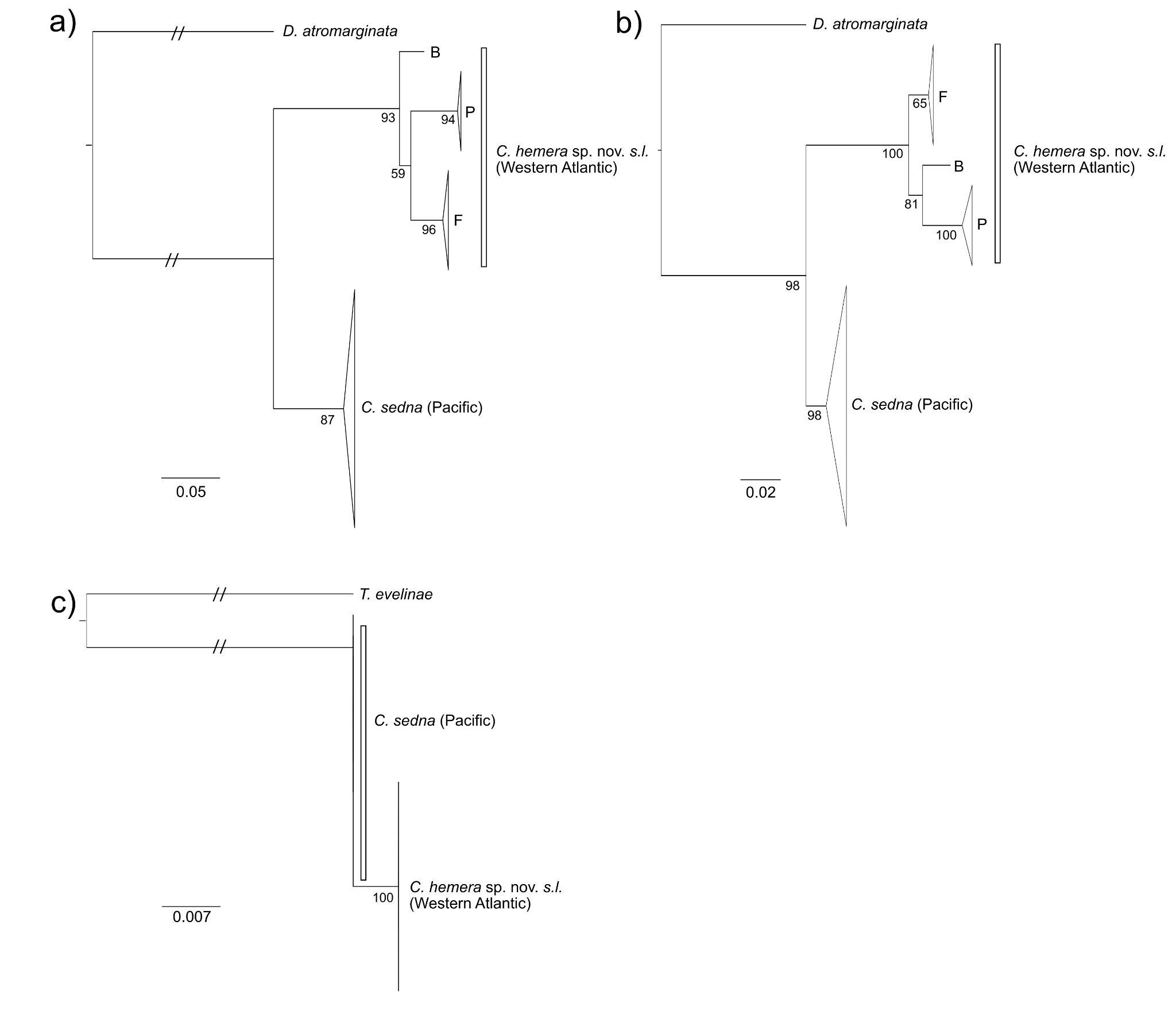


**Figure S1:** Maximum-likelihood phylogeny for *Chromolaichma* ‘*sedna*’ at a) COI, b) 16S and c) ANT, where triangles represent collapsed clades, hash marks indicate the branch was shortened by 50%, and bootstrap support is presented at nodes. Western Atlantic localities are indicated as follows: Bahamas (B), Florida (F) and Panama (P).


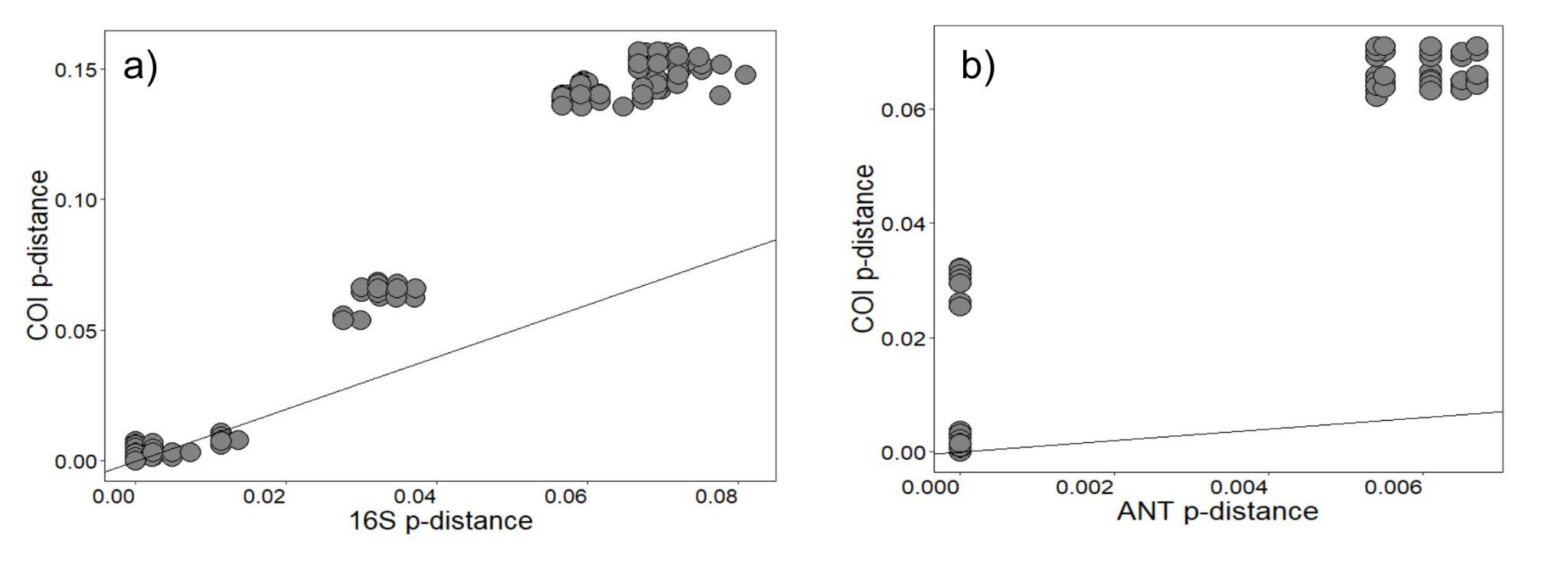
**Figure S2:** Comparing a) COI and 16S and b) COI and ANT pairwise distances among individuals. The solid line shows equal divergence between the two loci.

**Table S1:** Pairwise distances at COI for *Chromolaichma ‘sedna’*.


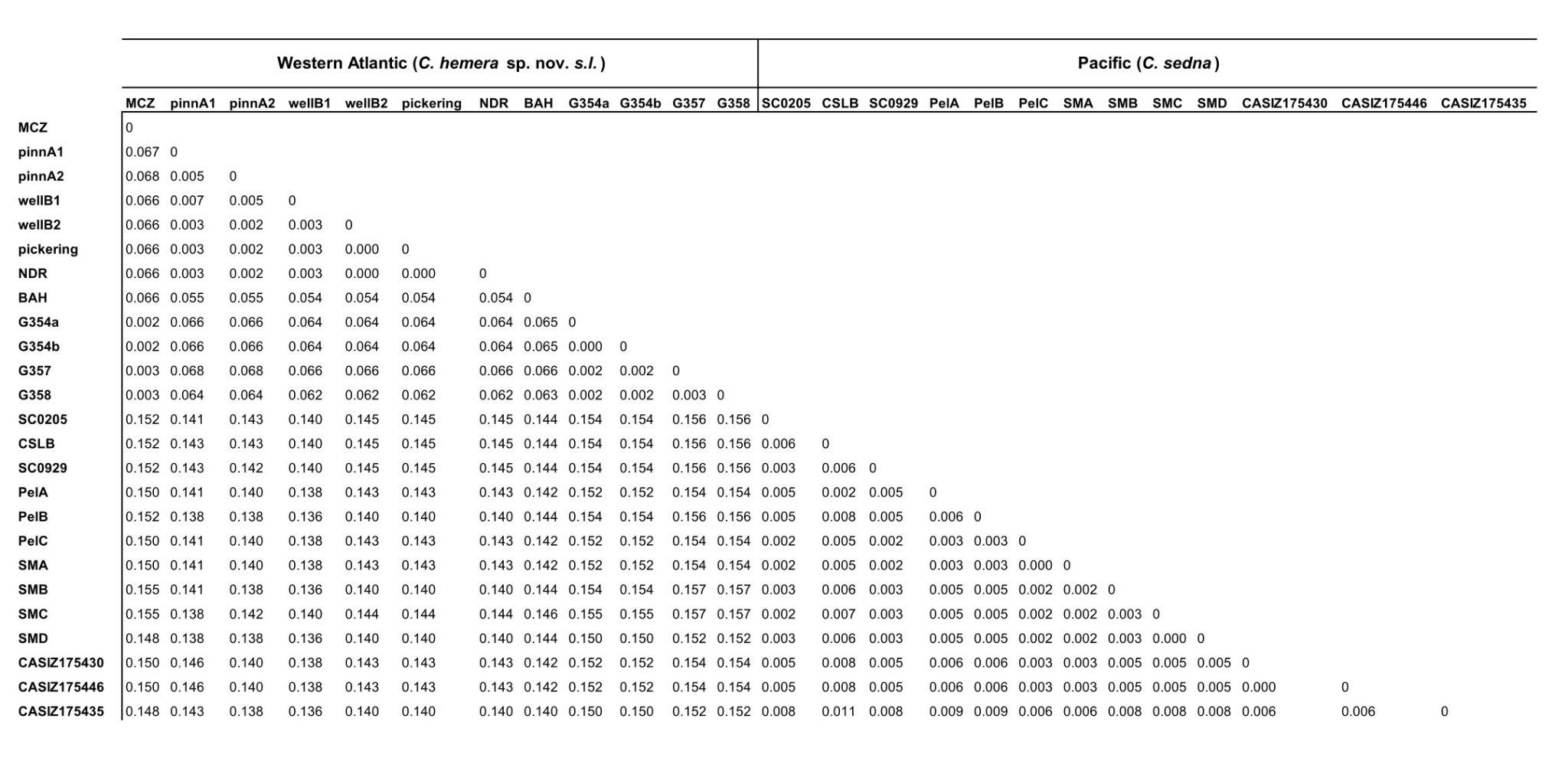


**Table S2:** Pairwise distances at 16S for *Chromolaichma ‘sedna’*.


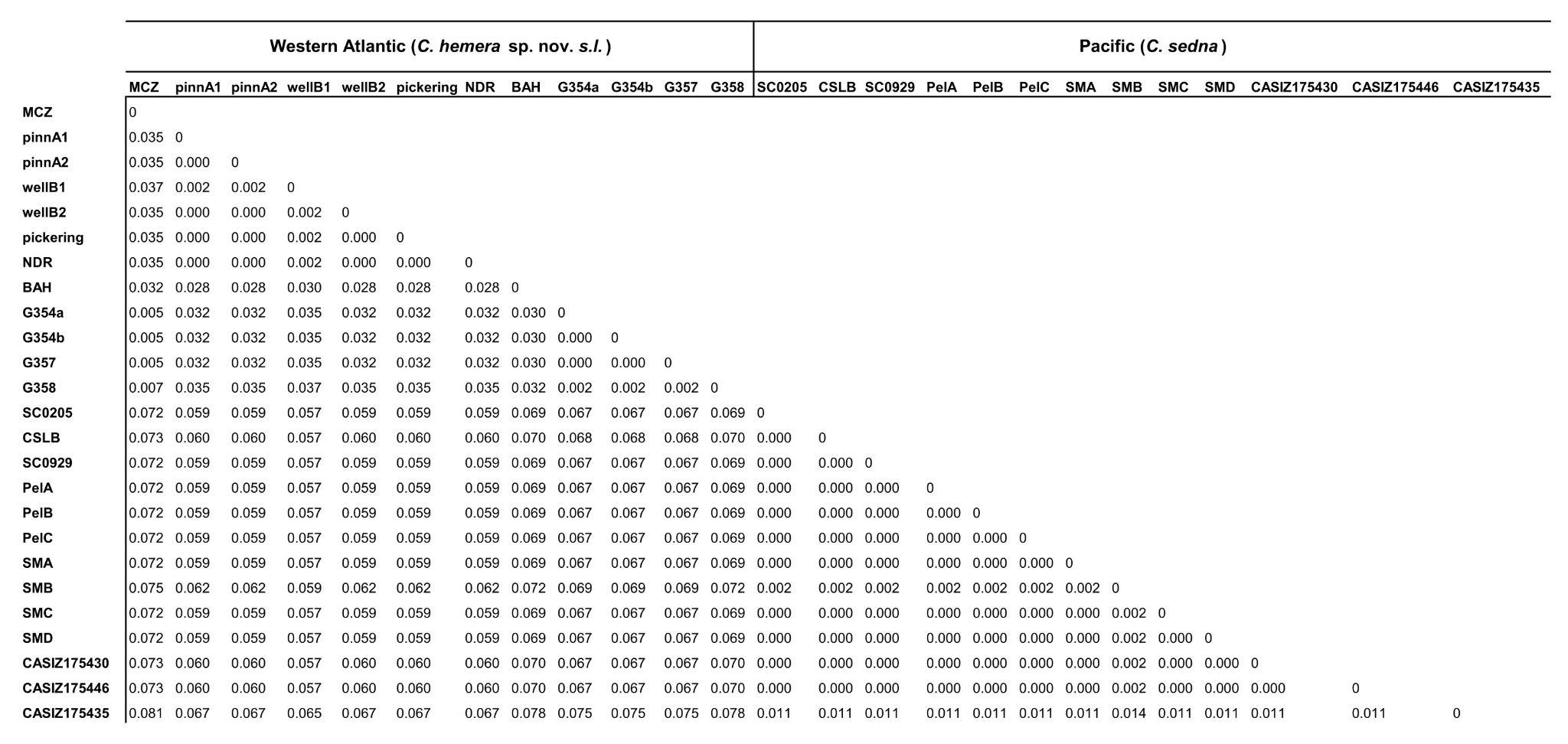


**Table S3:** Pairwise distances at ANT for *Chromolaichma ‘sedna’*.


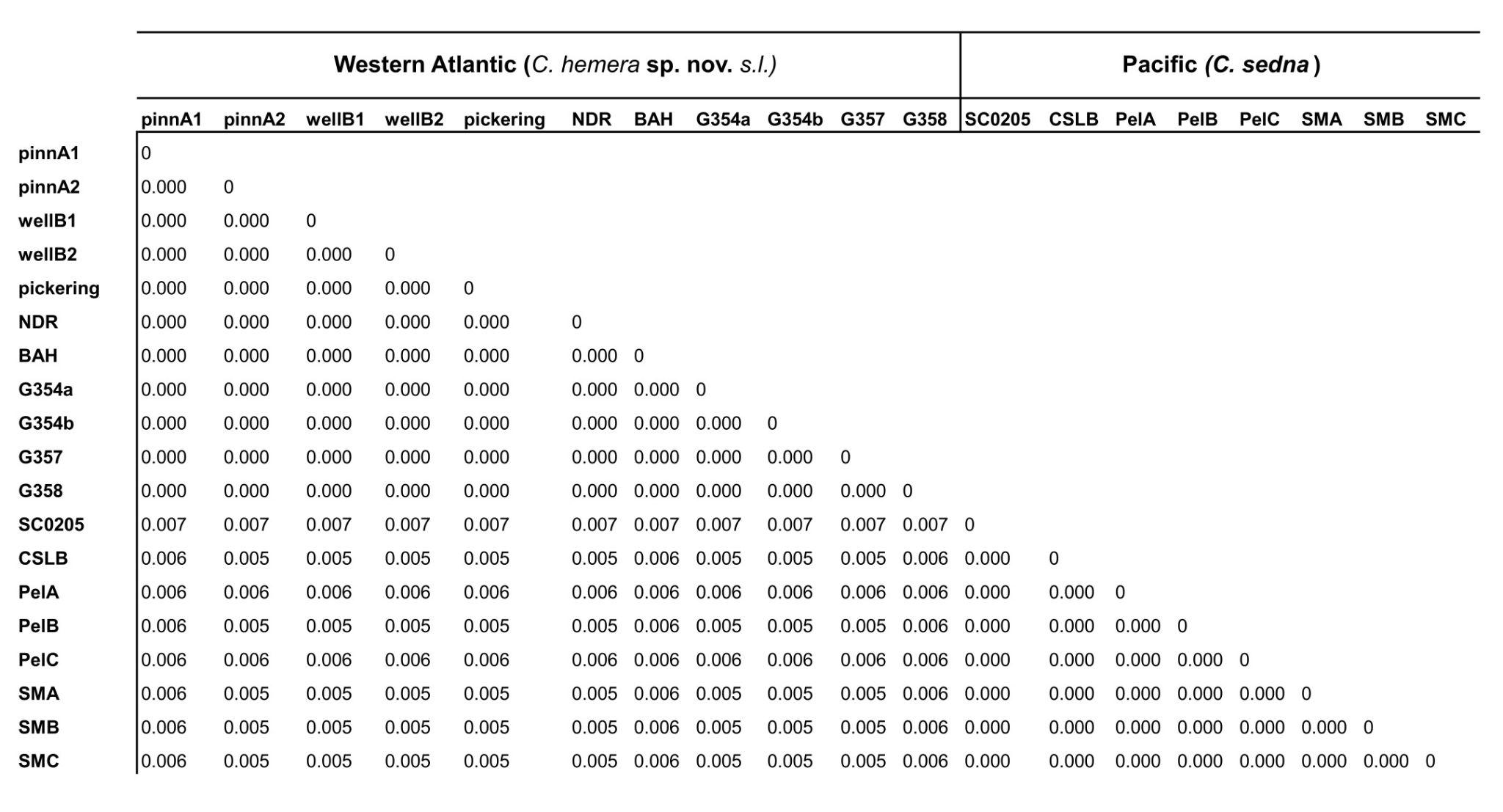

Supplement: Supplementary file 1 — Appendix S1 [file ECE3-14-e11014-s001.docx]
